# Socio-Cultural Reasons and Community Perceptions Regarding Indoor Cooking Using Biomass Fuel and Traditional Stoves in Rural Ethiopia: A Qualitative Study

**DOI:** 10.3390/ijerph15092035

**Published:** 2018-09-18

**Authors:** Mulugeta Tamire, Adamu Addissie, Susann Skovbjerg, Rune Andersson, Mona Lärstad

**Affiliations:** 1 Department of Preventive Medicine, School of Public Health, College of Health Sciences, Addis Ababa University, Addis Ababa P.O. Box 366 Code 1029, Ethiopia; adamuaddissie@gmail.com; 2 Department of Occupational and Environmental Medicine, Institute of Medicine, Sahlgrenska Academy, University of Gothenburg, Medicinaregatan 16A, 41390 Gothenburg, Sweden; mona.larstad@amm.gu.se; 3 Department of Infectious Diseases, Institute of Biomedicine, Sahlgrenska Academy, University of Gothenburg, Guldhedsgatan 10A, SE 41346 Gothenburg, Sweden; susann.skovbjerg@vgregion.se (S.S.); rune.andersson@gu.se (R.A.); 4 Department of Respiratory Medicine and Allergology, Institute of Medicine, Sahlgrenska University Hospital, SE 41390 Gothenburg, Sweden

**Keywords:** household air pollution, socio-cultural barriers, community perception, Ethiopia

## Abstract

Around three billion people in the world and 90% of the rural households in low-and middle-income countries are exposed to wood smoke with varying exposure levels and resulting health risks. We aimed to explore perceptions of the community towards indoor cooking and the socio-cultural barriers to bring change in Butajira, rural Ethiopia. We conducted a qualitative study involving ten separate focus group discussions with purposively selected members of the community and two key informant interviews with health extension workers. Content analysis was carried out using ATLAS.ti software. Participants reported the use of fuel wood and traditional three-stone cook stove to cook food. Economic status, lack of commitment, cultural views and concern along with safety and security issues were found to be barriers to change from traditional to cleaner methods of cooking. The community perceived wood smoke to have effects on their eyes and respiratory health, though they culturally viewed it as beneficial for postpartum mothers and newborns, avoiding bad smell and insects and in order to strengthen the fabric of their houses. Health education at community level is essential in order to bring about change in the cultural views and cooking behaviors focusing on opening windows and keeping young children away during cooking.

## 1. Introduction

Around three billion people in the world, and 90% of the rural households in the low- and middle-income countries utilize solid fuel for cooking and heating using traditional stoves [1,2]. Such inefficient cooking and heating practices produce high levels of household air pollution (HAP) including a range of health damaging pollutants, such as small soot particles that penetrate deep into the lungs, and carbon monoxide (CO) with exposures often far exceeding national standards and international guidelines. Exposure to HAP has been associated with a range of adverse health outcomes including chronic obstructive pulmonary disease (COPD), lung cancer, airway infections, tuberculosis and diseases of the eye in adults; low birth weight and, of particular concern, acute lower respiratory infections (ALRI) such as pneumonia amongst children less than five years of age [3]. Each year, close to 4 million people worldwide die prematurely from illnesses attributable to HAP, which is also responsible for an estimated 3.7% of the overall disease burden in low- and middle-income countries [2].

There have been global and local initiates and interventions including The Global Alliance for Clean Cook Stoves, to alleviate the problem of HAP due to solid fuel by supporting adoption of clean and safe household cooking solutions [4,5,6,7]. However, different research findings indicate the existence of resistance to accept some technologies and/or failure of achieving the desired objective in preventing the health problems in some communities [5,6,8].

Exposure to HAP and resulting health risks vary greatly in different types of societies because of cultural differences and household behaviors of cooking. It is also affected by age, sex and other socio-economic characteristics of the population under consideration putting the mothers and young children at higher risk in most cultures [3,9]. As a result, use of behavior intervention along with cook stove technologies and ventilation options have been used as intervention packages to address the problem of HAP and found to be effective [10,11].

Contextual understanding of the socio-cultural and other determinants of cooking behavior using a qualitative approach is crucial to plan appropriate interventions for the target community. To our knowledge, no prior study explored the existing practice and perceptions by involving men and women in rural Ethiopia. In the study, we sought to answer the following research questions:

- How do current traditional cooking practices affect HAP?
- What are the barriers for improvements?
- What does the community know about the health problems related to HAP?
- How strong is the intention of the community towards changing the traditional cooking to reduce HAP?

## 2. Methods

### 2.1. Study Approach

We conducted a descriptive qualitative study using the content analysis approach. Qualitative content analysis is a powerful method for systematically describing the meaning of qualitative data collected through interviews and focus group discussions [12].

### 2.2. Study Setting

The study took place at rural Butajira, Misrak Meskan district of the Gurage zone of the Southern Nations and Nationalities and Peoples Region (SNNPR), approximately 135 km south of Addis Ababa. One urban and nine rural kebeles (the lowest administrative level) of the area have been the site of the Demographic Surveillance System (DSS) of Addis Ababa University Rural Health Program, since 1987 [13]. The study focused on five purposively selected rural kebeles within the district.

### 2.3. Participants and Recruitment

Individual participants for the focus group discussions (FGDs) were chosen based on their permanent residence in the area for at least five years and are recognized as knowledgeable about their community irrespective of their sex. Accordingly, data were collected from members of the community at the five kebeles and health extension workers (HEWs) from two of the selected kebeles in two rounds in August 2016 and February 2018. Experienced researchers guided by field facilitators from Butajira Campus of Addis Ababa University visited the villages and purposively chose individuals recognized as knowledgeable about their kebele following a snowball technique, where the potential participants were asked at the initial contact if they know of others who could enhance a better understanding of the phenomenon under consideration.

### 2.4. Data Collection

A total of 10 FGDs, five males and five females, with members of the community in the selected villages comprising an average of seven participants in each session, were carried out. Besides, two key informant interviews with HEWs were conducted using a semi-structured guide with open-ended probing questions in the Amharic language. All the FGDs and interviews were carried at noise free places within the community and audio-recorded while appropriate notes were taken throughout the data collection. We summarized the main discussion points at the end of each FGD to the participants.

Review of the field notes and listening to the audio were used to see preliminary findings and to identify areas to be further explored, thus to determine the saturation point (information redundancy reached) by making a qualitative judgment by using saturation grid [14]. Two more FGDs, one from each sex, were conducted after the saturation of data was attended at the fourth kebele to determine real saturation was reached.

### 2.5. Data Analysis

All audio-records were transcribed verbatim and translated into English by an experienced translator for analysis. We provided both the transcriber and translator with a brief description about the research scope and objectives of the data to enhance their understanding of the subject matter. The transcripts and translations were cross-checked for consistency. Translated notes were read and re-read by the principal investigator with qualitative research experience to define categories and sub-categories guided by the objective of the study. Then a codebook was prepared and three of the authors (S.S., R.A. and M.L.) reviewed it before starting the coding. Content analysis was carried out using ATLAS.ti version 8.0 software (Scientific Software Development GmbH, Berlin, Germany) to code the transcripts based on the codebook. According to Schreier [12], qualitative content analysis approach gives the opportunity to choose aspects on which a researcher wants to focus during analysis. Following that, we used our interview guide as a deductive framework to build our coding frame and select main categories on which to focus. We used inductive approaches to create sub-categories, based on the meaning coming from the data.

### 2.6. Trustworthiness

#### 2.6.1. Credibility

According to Lincoln and Guba, prolonged engagement in the field, persistent observation, triangulation, checking interpretations against raw data, peer debriefing and member checking with a transparent design of coding and drawing conclusion from the data, were recommended to improve the credibility of the research result [15]. To ensure credibility the lead author stayed for two weeks in the area in two intervals to collect the data and to observe the actual context while the data were collected from both male and female community representatives, from five independent villages and health extension workers with place, time and person triangulation of the data. We had peer debriefing throughout and our interpretation and conclusions are from the raw data using direct quotes to elaborate the codes and categories.

#### 2.6.2. Transferability

This refers to the extent to which the results from one study are relevant to other contexts which in turn could be affected by how representative the sample is [16]. To this end, we had discussed how representative and information-rich samples were selected from the community itself and we provided descriptions that could be rich enough for other researchers to be able to make judgments about the transferability of our research findings to different contexts.

#### 2.6.3. Dependability and Confirmability

According to Bradley, dependability refers to the coherence of the internal process and the way the researcher accounts for changing conditions in the phenomena, while confirmability is about the extent to which the characteristics of the data, as posited by the researcher, can be confirmed by others who read or review the research results [17]. To fulfill these conditions, the lead author and two co-authors conducted codebook review and determined categories of description and agreement of the three authors was used as a dependability check. To establish confirmability, presentation of the categories at preliminary level to the research team was made to validate the process of analysis and findings by comparing with the quotes from the text. Another qualitative researcher recoded some of the meaning units into categories and coding-recoding evaluation agreement was achieved.

### 2.7. Ethical Considerations

Ethical approval was obtained from the Institutional Review Committee of the College of Health Sciences of Addis Ababa University and National Research Ethics Review Committee (NRERC, 3.10/168/2016), Ministry of Science and Technology, Ethiopia. All participants were asked to give consent for participation and audio recording before the commencement after explaining the objectives and importance of the study, whereas, confidentiality and anonymity were kept throughout the study.

## 3. Results

### 3.1. Participants’ Background

Table 1 shows the background characteristics of the participants. There were 69 FGD participants. The ages of the study participants varied from 30 to 65 years (mean 43 years). All of them were married. All the male participants were farmers while the females were house-makers. Eight of the mothers worked as merchants on local market days. The total family size ranged from three to a maximum of eleven with an average size of seven including relatives permanently living with the family. The majority was Muslim religion followers (71%), attended elementary school (58%) and almost half had tin-type house (houses made using corrugated tin sheet) for main living while the rest still use tukul (traditional thatched roof hut). In addition, two health extension workers were involved in the in-depth interview.

We described the findings of the study using the main categories with continuous text and organizing by categories. Table 2 shows detail sub-categories.

### 3.2. Current Cooking Practice

This main category describes the fuel types and sources, types of stoves used, cooking places, the housing conditions with the ventilation types and who takes the responsibility of cooking and who else are exposed during the cooking process. The participants clearly depicted that wood was the major type of fuel to cook food in the community. However, crop residues, cow dung and straw were also used during the dry seasons. The use of charcoal was not common in the area.

“We use wood. We also use stem of maize, dung, branches or leafs of trees and we rarely use charcoal in this community (Noise from the group saying we do not use charcoal). … Some family members who have tin house may rarely use charcoal to prepare tea, not for cooking food. Actually, wood is the main fuel source for us.”

(Female FGD: WRP2)

Regarding the sources, the absence of forest in the nearby area forced them to plant trees on their own lands or even to buy the fuel in rare, worst situations. Collection of the crop residues and straw during the dry or harvesting season was usually from their farm or leftovers from the others’ in their village. They also reported that cow-dung cake could be prepared in different forms at home or collected from the field, though some claimed that they started using it as manure instead.

“There are some, who have their own trees on their land and there are others, who plant new trees by using their farmland, which has been used for other crop production. Some even do not have enough extra land to plant new trees and there are still others, who buy the wood in some occasions.”

(Male FGD: WRP6)

**Figure 1:**
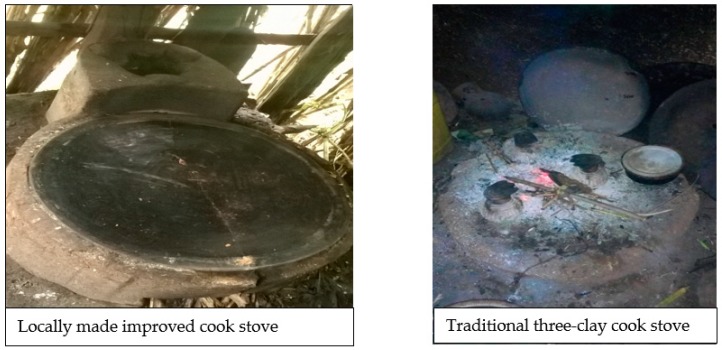
Photos of locally made improved cook stove and traditional three-clay cook stove.

Overall, the community used traditional three-clay cook stoves to prepare food, but some community members also used locally-made improved cook stoves, provided by a non-governmental organization for a low price (Figure 1). However, the use of improved cook stoves across the kebeles varied from only 15 to 200 households out of an average 600 households per kebele as stated by the health extension workers. There were also some community members using both types of stoves at a time claiming that the improved one was not suitable to use for all-purposes. They mentioned that to cook cabbage, wat (Ethiopian stew or curry) and prepare coffee they still used the traditional one, while they had the improved one for bread and injera (the national dish).

“Some of us have improved types of stoves for injera (the traditional Ethiopian food) but we commonly use the traditional three clay stoves and open fire to cook other things.”

(Females FGD: DGP4)

The place to cook food was not uniform at community level. The participants estimated slightly more than half the community members had a separated kitchen to use while the rest cooked inside the main living house where they slept and even shared with other domestic animals. Whether they had a separated kitchen or cooked inside the living house, the traditional stoves were located near the center of the house in a circular space made of mud to protect distribution of ash to the rest of the house. The location of improved stoves was always near the walls of the houses, not in the center, as it needs to be supported off the ground. The community members also indicated that cooking outside the house was uncommon in the area. However, they could cook outside when they have special occasions or ceremonies demanding cooking more food than the usual. In these occasions, either they constructed temporary shelters or dug the ground to facilitate the burning process.

“Not all have the same type of kitchen if we discuss about the community. Some have the ability to construct separated kitchen and to have an improved stoves while others cook in the same place or in the main living house and share the house with the animals. We do not cook outside.”

(Male FGD: SBP2)

Regarding the housing types and ventilation, the participants mentioned there was a shift from the traditional tukul houses to tin houses for the main living house. Once the household constructed tin houses for the living purpose, indoor cooking was not practiced there for the smoke was viewed not well for the roof color. They indicated that nearly all the houses used for the kitchen were the traditional tukul houses. There were three alternatives mentioned as means of ventilation used in the cooking areas: having only the door, one additional window, or permanent small openings near the end wall. When asked about the behavior of opening existing windows during cooking, they said they might open it when the level of smoke got very high and they found staying in the room was not tolerable. They indicated that opening a window whenever cooking was rare if the smoke level was at the usual level.

“…we do not cook in the main living house of the modern type. We use the tukul house for cooking but there are a few who also constructed another separated kitchen made of tin roof.”

(Male FGD: WR P4)

*“We do not open it during the night times* (others mentioned it is not safe to open windows at night) *but at daytimes we may open it for a while when it becomes very smoky. We rarely open the windows during the nights.”*

(Male FGD: DRP6)

Mothers were responsible for cooking food for the whole family and girls took part in supporting the mothers after school. FGD participants, from both male and female groups, reflected that children could stay with the mothers during cooking even in the houses with the separated kitchens. They also added that it was not rare to see the whole family spending time in the kitchen to eat meals and have coffee time. Two participants pointed out that their family used to stay in the kitchen all the day and only go to the main house for sleeping unless guests or strangers were around.

“We (women) are responsible for cooking food and for all the activities in the house. Children will get to the kitchen with us. Otherwise, we cannot go here and there to take care of the children and cook food in different places. The children stay with their mother in the kitchen.”

(Female FGD: WR P7)

### 3.3. Reasons for Traditional Indoor Cooking

The participants listed different reasons why the community continued to use the traditional ways of indoor cooking with biomass fuels and traditional stoves. Under this category, we also included the reasons why poor ventilation predominated and why mothers did not open the available windows during cooking.

The major barrier mentioned across all focus group discussions was the current economic status of the community that hindered their ability to afford a separated kitchen, improved cook stoves and a better type of house they needed to construct. Connected to the economic status, the community believed that small living houses were not appropriate to the improved cook stoves as the space was not enough to accommodate it in addition to the family and their animals.

“… We have limited ability to change it (stove) and we cannot even separate the kitchen from the main house. Some have even small house and there is not enough space to install the modern stoves. It is because we have problem with affording ability of the family and the economic status that we are using the traditional stoves.”

(Male FGD: SBP6)

However, two participants in the male FGDs stated that a lack of awareness about the effects of exposure to the biomass smoke was the main barrier for the community not to try to change the situations and give priority to the issue. They argued that with greater awareness, it would have been possible to invest in changing the stoves and kitchen type with priority over other demands to fulfill the urgent needs and to keep social status. Connected to this, the moderator also asked how many community members had a mobile phone, they mentioned nearly all had at least one at household level.

“They may find mobile phone at least at family level. Some buy it because the students persuade them to listen to music or FM radio or due to peer pressure. If you ask them to buy stove using the money, I do not think anybody do that.”

(Male FGD: SBP6)

Participants also mentioned the issues of safety and security as reasons to cook inside the house and for the absence or not opening the existing windows during the night for better ventilation. This was linked with fire hazards especially during the dry season, which hindered them from cooking outside and they were afraid thieves would break and enter into the house if the windows were open.

“In the dry season there is wind and everything is dry. So, there will be high risk of fire hazard as everything including grasses could burn and the fire could get to the main living house which is even more dangerous.”

(Male FGD: SB P1)

“Let alone opening, even most of the separated kitchens do not have windows for a security reason. As there will be animals and stored cereals/grains, they fear that someone can break the window and steal it. Some do not open windows during the night as they suspect it is not safe.”

(Male FGD: SB P3)

Lack of alternative fuel types, especially electricity, was mentioned in the discussion as a reason for using biomass and the traditional stoves. They emphasized that even the locally produced improved cook stove had a problem of smoke although at a reduced level. All agreed that if there was enough electric power a shift was likely towards use of modern stoves for cooking.

“There is no electricity and we have no other option than the wood and the smoke. We know it has effects but we have to cook and survive.”

(Female FGD: MM P4)

The participants agreed cooking outside was not a tradition of the community. There were also traditional views and myths, which could favor the continuation of cooking inside the house all the year. Community members believed that cooking or eating outside could lead to disease.

*“There will be risk of mitch* (Amharic; literally means falling sick from exposure to sunlight with food) *as there will be a problem due to smell from the food and it causes disease when sweating during cooking outside”*

(Female FGD: DGP3)

Mothers in the FGDs mentioned that the type of food cooked in the area were reasons for not changing the traditional ways. They thought it was not conducive to cook injera outside because of the wind during the dry seasons as it affects its texture. Even those who have the improved cook stoves did not find it appropriate to cook wat (Ethiopian stew or curry) though the design was to use it for both purposes.

“I have an improved cook stove for the traditional injera but for other types of food including wat or other cabbage I use the traditional three clay cook stove.”

(Female FGD: DG Px)

### 3.4. Perceived Benefits of Wood Smoke

This category describes the perceptions of the community regarding the benefits of the smoke in the house. Existence of smoke in the house was believed to heat the house during the rainy season and night times, to avoid smell from animals (dung), strengthen and preserve the fabric of the traditional tukul houses and to control pests.

“There are some types of flies that can enter into the living houses and they cannot stay in the house if there is smoke even in small amount. And, it also heats the room and that is the advantage of the smoke.”

(Male FGD: DG P6)

Some participants pointed out that a traditional tukul house was not a house unless inside burning and smoke release was there, while others added that smoke was a natural way of conditioning a house.

“Yes, there should be some smoke in the house on a daily base because the cattle live in the house and there could be smell and we use the smoke to avoid it (smell). It is not good without smoke. After cleaning the house in the morning, we smoke it to refresh. We need existence of smoke at least at small level and we can say it has benefit in that aspect.”

(Females FGD: MM P3)

The participants also added that smoke was important from the cultural point of view of the local community explaining it shows a better status of the household and cooking skill of the wife. In this culture, smoke was perceived as good for a mother newly giving birth and her baby especially in the first months.

“We like smoke in the rainy season and even some community members may think this village belongs to the poor if there is no smoke visibly coming out in the village. When there is good emission of the smoke from the houses of the village the community or any person passing by will think that the village dwellers are rich and the wife is good at cooking. Therefore, we need to burn fire and smoke our houses because of the culture.

(Female FGD: MMP3)

One of the health extension workers added that culturally the community viewed the smoke as a good thing. According to her experiences, even those who had a tin house and did not cook inside the living house often were keeping the mother and her newborn in the tukul house and burned wood inside.

“The community believe that the heat from the smoking house will help the mother and the newborn to become fatter and healthier…To fulfill that, the husband is expected to avail adequate wood even by cutting big tree to burn continuously whether there is cooking or not, to keep the house warmer at least for the first two months”.

(HEW: WB)

### 3.5. Perceived Risks of Wood Smoke

All the participants across the FGDs repeatedly pointed out the effects of the smoke mainly on the eyes of the women who are cooking. Female FGD participants remembered the effect on the eyes as tiresome and the most discomforting effect of using firewood. Participants also frequently mentioned respiratory health problems, particularly a cough, nose irritation, shortness of breath and the effects on the lungs. Despite the fact that the FGD participants could not specify the details there were some participants who perceived the smoke was not good for their general health.

I think the main problem is related to the mothers and their eyes. It seems they are crying when they cook. It can also lead to lung problem and asthma as the problem increases. I think the smoke makes them not able to breathe properly and it is not good for their respiratory bodies.

(Male FGD: DB P1)

Tuberculosis among mothers and pneumonia in children were also reported as risks resulting from wood smoke, and health extension workers confirmed that pneumonia was the leading cause of morbidities among children in the areas.

“We commonly found pneumonia in the children and we know that it is because of the pollution. The problem is very common even in the infants as the mothers keep the children with them and they are highly exposed to the smoke during the postpartum periods because of indoor burning.”

(HEW: WB)

Some participants also thought their aesthetic value could be affected because of the smoke and their exposure during cooking. Although most perceived that smoke was good to avoid the bad smell from animals, another group believed that the smoke itself had a smell, which could stay on their clothes and body and decreased their hygiene status.

“Firstly, it affects the eyes as it has been well explained before and it has smell left on the clothes that the clothes smell smoke after you leave the house with smoke. It also changes the color of the house and other materials inside the house even including the face of individuals. I can also say that smoke is not good for the people and it has many disadvantages.”

(Male FGD: DRP4)

### 3.6. Intention to Accept Changes

Regarding the intention to change the existing tradition of indoor cooking using biomass and traditional stoves, the general answer was ‘yes’ from all participants. However, some participants argued that the overall concern of the community was low because of low awareness and other priorities. There were different priority issues in the community starting from fulfilling the basic need to attaining the social status. Others also link this with poor education and lack of enough knowledge regarding the long-term effects of using this cook stove. One participant from male FGD stated that:

*“…there are also social responsibilities where we are expected to make contributions like for Edir* (Traditional association like insurance during death). *We may contribute or go to hospital to ask someone who is sick rather than buying the stove. They are not the things, which are critical for change in our life but they are instead social and traditional aspects. If you ask me, the stove could bring more change in my life than the other social processes. It is after you save yourself that you can help others but that is not true here in traditional community we give priority to the social norms more than our personal lives.”*

(Male FGD: WR P6)

Overall, the community needed changes and all mothers using improved cook stoves had positive attitudes towards using them and spoke of the benefits on the reduction of the amount of wood used and time needed to cook food. They also stated that the level of smoke emitted and the exposure to flame and direct fire burning was low compared with the traditional ones.

“The consumption of wood using the stove type we have is huge but I saw that those mothers who have the improved cook stove use smaller amount as once it is hot the stoves keeps the heat for longer time. The mothers cooking on the new stove don’t suffer from sweating as the fire does not reach them directly.”

(Female FGD: SBP2)

However, participants from two of the involved kebeles indicated that some mothers did not like the new stoves and having to stand while cooking. There were even mothers who stopped using the stove after testing and others gave it to their relatives. Their reason for this was that the mother could accomplish other activities including breast-feeding her child while cooking with the traditional stove. There was argument during the focus group discussion on the issue but those who were using the new stove did not accept the justification.

*“Some community members who had good understanding used it* (improved cook stove) *since then and others even keep it until now or gave it to their relatives, as they were not interested to use it. They said they did not like standing and waiting to finish cooking.”*

(Male FGD: SB P1)

The health extension workers also shared that most mothers in their catchment area learned to know the benefits of the improved cook stoves by observing those using them and started to request them for themselves.

“Some of them use it and others were also requesting us after looking from them but we could not provide it as the price also goes up. Still some mothers complain about it because they did not get it. They thought I did not give them the chance and still they need to have it.”

(HEW: SB)

A male FGD participant also raised the issue of shortage of firewood in the area and its effects on the productivity of the farming land when planting trees to overcome this. All the group members agreed on the point and indicated their intention to change to the stoves with less wood consumption in the future.

“Previously, we used to get trees as heritages from our parents or even ancestors but now there are a limited number of such things so we are expected to plant on our farmlands. The problem by now is, as the trees have effects on the land use of the neighboring farmers there is a local law, which prohibits planting trees very near to others’ farm. Therefore, we face problem because of it. In one way, it is not possible to get in the center of the farm and on the other hands; we cannot get near to the others so that it will be problem for the future.”

(Male FGD: WRP6)

### 3.7. Suggested Solutions for Future Interventions

We finally asked the participants to give suggestions on the way forward regarding interventions to address the problem of indoor cooking using biomass fuel and traditional cook stoves. Under this dimension, we identified three sub-categories from their discussions, namely: Reaching the masses to create awareness; ease of use and maintenance of the technologies; and affordable cost and best seasons to supply.

The community members emphasized the need for awareness-creation on the effects of biomass and traditional cook stoves and any type of interventions planned at community level. They indicated that the community might not consider the problem as serious and prioritized the issue to invest or accept any intervention.

“What (intervention) we need in the future is what involves the whole community. Still there is commitment problem because of lack of awareness and should be addressed.”

(Female FGD: WRP4)

Mentioning a previous failure to use and maintain a solar energy lighting system, the participants also suggested that any technology to be provided to the community should be easy to use and maintained locally.

“Now we use battery for lighting. We used to buy and use solar lighting, which has three bulbs but it became not functional after some time and when we take for maintenance, it was very expensive as they request us to pay 250 ETB (9 USD). After two or three weeks’ time of maintenance, it became not functional then we stopped using it. We do not need to suffer in this way in the future”.

(Females FGD: DG P3)

Regarding the cost and seasons to supply improved cook stoves, they pointed out that the community has different needs and priorities and some might not even afford the minimum cost of 100 Ethiopian birr (below five USD). They recommend that supplying in the harvesting season, when they have a better income, could help the farmers afford the cost while some also suggest availing payment at different phases before and after the supply.

“The community may buy, not all of them but most. It is good if it is better quality and soon after harvesting season as there is no other income source for the farmers here. Our community has a tradition of buying such type of instruments using payment made at intervals.”

(Male FGD: SB P7)

## 4. Discussion

Like other rural areas in different developing countries, biomass was the only fuel option in the community. Previous studies also reported that nearly all rural communities used biomass fuel for cooking [8,11,18]. However, our finding regarding not using charcoal as a fuel source by the community for either cooking or heating was inconsistent with other findings reporting its continuing use by many in rural communities [19,20,21]. Despite a recent study in 2011 [20] in the same region claiming no use of improved cook stoves, our evidence shows improvements in the use of locally produced cook stoves by the community. During the discussion, the community members reported that a local NGO, which took the responsibility of distributing the cook stoves, did not sustain the supply while there was demand. This could be considered as a lack of an enabling environment for the community to adopt and sustain the changes.

The community used biomass, mainly wood, regardless of the type of stove. A shortage of firewood and absence of forests in the area could be used as an opportunity to shift the biomass fuel use to cleaner energy options, particularly solar energy in the area. This could be feasible due to Ethiopia being listed as one of the countries with the highest solar resources and in the long run, the cost of investing in such alternatives was confirmed to be of the same order as cooking with conventional fuels [22]. However, solar cooking in rural area is still a critical and not well-developed technology especially in the developing world. Eswara and Ramakrishnarao noted in their study that high initial cost of investment in the use of solar energy was a hurdle for small-scale food processing [23].

The participants reported that more than half the community in the area had a separated kitchen in which to cook food. Obviously, there have been changes in the housing conditions and cooking in separated houses in the last two decades illustrated by comparing with a previous study which reported 95% of the inhabitants in the same rural area had one single room for living and cooking [24]. Nevertheless, the use of traditional stoves and tukul houses with poor ventilation for cooking in the separated kitchens subjected the mothers to similar risks of developing different health problems as cooking in the main living house. To this end, previous studies identified a higher occurrence of cataracts and different respiratory symptoms, including chronic obstructive pulmonary disease and reduction in the lung function among mothers exposed to biomass smoke [3,25,26]. As expected, mothers and young children, including the infants, were at highest risks whether the household used the main living house or a separated kitchen for cooking [9].

Our findings showed that the availability and practice of ventilation in the cooking area was poor, as the participants mentioned either the mothers not opening existing windows regularly or there were no windows at all. A study from China also reported the existence of never opened windows during the winter period, but ventilation was significantly improved after delivery of health education [10]. It has been recommended that the presence of at least one or more windows in the kitchen area is critical, including adequate size and cross ventilation to facilitate natural ventilation [27].

A lack of economic resources was the leading barrier to change the existing tradition of cooking and the housing conditions as stated by the participants of the study. It is obvious the economy of the household could determine the type of house and the possibility to invest in alternative fuels and improved cook stoves. Other studies from Uganda and Nepal also identified financial considerations as the most influential factors related to improved cook stove acquisition and use [11,28]. A report from another region in Ethiopia also found that women were willing to change cooking practices but were unable to afford cleaner fuels or improved stoves [29].

The absence of prioritizing health benefits over other social and personal needs was noticeable. In our study, some participants linked this with a low level of education and lack of enough awareness regarding the long-term effects of exposure to household air pollution. This was also consistent with responses in the Nepalese study [28]. The fear of fire hazard and being afraid of thieves were the safety and security reasons for not cooking outside and having open windows. We were unable to find similar reasons in other studies. The tradition of cooking inside a house by considering a smoky indoor air as a natural event was consistent with the Nepalese study [28].

The community perceived that wood smoke had benefits for strengthening and preserving the tukul houses, to avoid bad smells from the cows and to control pests. Moreover, they perceived that it was good for the health of a mother and newborn during the postpartum period and had a positive cultural value of keeping the house smoky as a sign of existence of inhabitants and indicating good social status. This could also result in hindering the possibilities of shifting the traditional cooking to cleaner energy in the future. A systematic review showed a similar reason of smoke protecting against insects being a barrier of adoption of cook stove interventions [30].

All participants perceived that HAP was risky for the eyes and respiratory health problems. From the discussions, they specifically mentioned almost all respiratory symptoms, pneumonia, tuberculosis, and effects on lungs including asthma. This is consistent with previous findings where mothers were aware of the health effects of wood smoke in the respiratory and eye health problems [28,29,30,31]. We included and asked the men/husbands in our study whose health they believed to be more affected by the smoke–they believed it is the women’s health.

Overall, we found the community had a positive attitude towards using improved cook stoves and intended changes in spite of the barriers and their perception towards the benefits of the wood smoke. Participants also reported on the shortage of firewood in the area and using the farmland to plant trees to solve it. This could be taken as a missed opportunity to implement large-scale cook stove intervention to address the problem of household air pollution in the area as it could facilitate the acceptance of the intervention by the community [32]. A qualitative study in Kenya also found that women preferred stoves, which helped them use less fuel [33].

This is a qualitative study in a specific ethnic group thus the study cannot be generalized to the national level, as the housing, tradition of cooking and related community beliefs and perceptions are culture specific. However, we included both sexes and health extension workers from different areas of the locality to increase the transferability of the findings. The lead author had repeated exposure to the community in the area and all authors had visited some homes before the data collection, which helped improve understanding of the situation and gain consensus on the meanings.

## 5. Conclusions

The facility of a separate kitchen and use of improved cook stoves by some community members did not avoid the use of biomass as the only fuel source, thus, it might not reduce health risks from HAP for the women in the area. Economic status, lack of commitment, cultural views and concern along with safety issues were found to be barriers to change the existing traditions of cooking. The community perceived wood smoke as beneficial though they knew it has negative effects on their eyes and respiratory health. Shortage of firewood in the community and their positive attitude towards improved cook stoves were good opportunities to induce demand to shift the energy use and plan stove interventions. Health education at community level is needed to stop the culture of exposing postpartum mother and newborn to wood smoke, to bring behavioral change in opening windows during cooking and create awareness on keeping young children away from cooking places.

## Figures and Tables

**Table 1:** Background characteristics of focus group discussion participants.

Characteristics	Number (*n*)	Percent (%)
**Sex**		
Women	37	54
Men	32	46
**Education**		
No school	24	35
Can read and write without attending school	3	4
Elementary school	40	58
High school	2	3
**Religion**		
Muslim	49	71
Orthodox Christian	14	20
Protestant Christian	6	9
**Type of living house**		
Tukul	35	51
Tin house	34	49
Mean family size (Range)	7 (3–11)	
Mean age (Range)	43 (30–65)	

**Table 2:** Main and sub-categories with response summaries.

Main Categories	Sub-Categories	Response Summaries
Current cooking tradition	Fuel options and sources	Mainly wood from their own farm/land
	Types of stoves	Traditional three-clay cook stoves
	Cooking places	Inside the living house or separated kitchen
	Housing and ventilation	Do not open window during cooking
	Cooking responsibility	Women cook but girls could help them after school
	Wood smoke exposure	Mothers and small children stay more in the kitchen
Reasons for traditional indoor cooking	Types of foods prepared	Cooking injera or bread outside is difficult
	Economic status	Cannot afford for the improved cook stove
	Lack of awareness	Poor awareness on health risks at community level
	Safety and security	Risk of fire hazard and theft during the night time
	Weather problems	Rain during winter and wind break in the summer
	Type of house	Some houses are small in size for the family
	Lack of alternative	Lack of alternative fuel option than biomass
	Tradition and Myth	Being exposed to sunlight with food causes disease
	Priority issues	Priority from basic need to keeping social status
Perceived benefits of wood smoke	Avoid bad smell	Natural refresher for a house
	Vector control	Avoids flies/mosquitoes from entering the house
	Strengthen the house	Gives longer life span for traditional tukul houses
	Cultural benefits	Good for the mother and newborn
	Heating the room	Heats the room during rainy seasons
Perceived risks of wood smoke	Respiratory health problems	Cough, nose irritation, shortness of breath and lung problems
	Eye problems	Eye irritation/tearing
	Effect on aesthetic value	Sweating, smell on the clothes and hygiene problem
	Other problems	Tuberculosis and affect general health status
Intention to accept changes	Community concern and priority	Less concerned and give priority to other needs
	Attitude towards using new stoves	Positive attitude
	Fuel shortage to favor any change	Shortage of fire wood could favor the changes
Suggested solutions for future interventions	Reaching the mass	Interventions at community level
	Ease of application and maintenance	Prefer locally made technology with ease of application and maintenance
	Affordable cost and best seasons to supply	Provide with low cost at harvesting season

## References

[1] Desai M.A., Mehta S., Smith K.R. (2004). Indoor Smoke from Solid Fuels: Assesing the Environmental Burden of Disease at National and Local Levels.

[2] WHO Household air pollution and health (Factsheet). http://www.who.int/en/news-room/fact-sheets/detail/household-air-pollution-and-health.

[3] Gordon S.B., Bruce N.G., Grigg J., Hibberd P.L., Kurmi O.P., Lam K.-B.H., Mortimer K., Asante K.P., Balakrishnan K., Balmes J. (2014). Respiratory risks from household air pollution in low and middle income countries. Lancet Respir. Med..

[4] United Nations Foundation About the Global Alliance for Clean Cookstoves [Internet]. http://www.unfoundation.org/what-we-do/campaigns-and-initiatives/cookstoves/.

[5] Mortimer K., Ndamala C.B., Naunje A.W., Malava J., Katundu C., Weston W., Havens D., Pope D., Bruce N.G., Nyirenda M. (2017). A cleaner burning biomass-fuelled cookstove intervention to prevent pneumonia in children under 5 years old in rural Malawi (the Cooking and Pneumonia Study): A cluster randomised controlled trial. Lancet.

[6] Romieu I., Riojas-Rodriguez H., Marron-Mares A.T., Schilmann A., Perez-Padilla R., Masera O. (2009). Improved biomass stove intervention in rural Mexico: Impact on the respiratory health of women. Am. J. Respir. Crit. Care Med..

[7] Smith K.R., McCracken J.P., Weber M.W., Hubbard A., Jenny A., Thompson L.M., Balmes J., Diaz A., Arana B., Bruce N. (2011). Effect of reduction in household air pollution on childhood pneumonia in Guatemala (RESPIRE): A randomised controlled trial. Lancet.

[8] Barstow C.K., Nagel C.L., Clasen T.F., Thomas E.A. (2016). Process evaluation and assessment of use of a large scale water filter and cookstove program in Rwanda. BMC Public Health.

[9] Bruce N., Rehfuess E., Mehta S., Hutton G., Smith K., Jamison D.T., Breman J.G., Measham A.R., Alleyne G., Claeson M., Evans D.B., Jha P., Mills A., Musgrove P. (2006). Indoor Air Pollution. Disease Control Priorities in Developing Countries.

[10] Cheng Y., Kang J., Liu F., Bassig B.A., Leaderer B., He G., Holford T.R., Tang N., Wang J., Jian H. (2015). Effectiveness of an indoor air pollution (IAP) intervention on reducing IAP and improving women’s health status in rural areas of Gansu Province, China. Open J. Air Pollut..

[11] Martin S.L., Arney J.K., Mueller L.M., Kumakech E., Walugembe F., Mugisha E. (2013). Using formative research to design a behavior change strategy to increase the use of improved cookstoves in peri-urban Kampala, Uganda. Int. J. Environ. Res. Public Health.

[12] Schreier M. (2012). Qualitative Content Analysis in Practice.

[13] Shamebo D., Sandstrom A., Wall S. (1992). The Butajira rural health project in Ethiopia: Epidemiological surveillance for research and intervention in primary health care. Scand. J. Primary Health Care.

[14] Brod M., Tesler L.E., Christensen T.L. (2009). Qualitative research and content validity: Developing best practices based on science and experience. Q. Life Res..

[15] Lincoln Y.S., Guba E. (1985). Naturalistic Inquiry.

[16] Krippendorff K. (2004). Content Analysis: An Introduction to its Methodology.

[17] Bradley J. (1993). Methodological issues and practices in qualitative research. Lib. Q..

[18] Magitta N.W.F., Walker R.W., Apte K.K., Shimwela M.D., Mwaiselage J.D., Sanga A.A., Namdeo A.K., Madas S.J., Salvi S.S. (2018). Prevalence, risk factors and clinical correlates of COPD in a rural setting in Tanzania. Eur. Respir. J..

[19] Musafiri S., van Meerbeeck J., Musango L., Brusselle G., Joos G., Seminega B., Rutayisire C. (2011). Prevalence of atopy, asthma and COPD in an urban and a rural area of an African country. Respir. Med..

[20] Yirsaw B., Hammed S., Araya A. (2011). Household fuel use and acute respiratory infections among younger children: An exposure assessment in Shebedino Wereda, Southern Ethiopia. Afr. J. Health Sci..

[21] Apte K., Salvi S. (2016). Household air pollution and its effects on health. F1000Research.

[22] Batchelor S., Brown E., Leary J., Scott N., Alsop A., Leach M. (2018). Solar electric cooking in Africa: Where will the transition happen first?. Energy Res. Soc. Sci..

[23] Eswara A.R., Ramakrishnarao M. (2013). Solar energy in food processing—A critical appraisal. J. Food Sci. Technol..

[24] Kumie A., Berhane Y. (2002). Crowding in a traditional rural housing (“Tukul”) in Ethiopia. Ethiop. J. Health Dev..

[25] Bihari V., Iqbal S.M., Srivastava L.P., Kesavachandran C., Siddique M.J. (2013). Lung function impairment in women exposed to biomass fuels during cooking compared to cleaner fuels in Uttar Pradesh, India. J. Environ. Biol..

[26] West S.K., Bates M.N., Lee J.S., Schaumberg D.A., Lee D.J., Adair-Rohani H., Chen D.F., Araj H. (2013). Is household air pollution a risk factor for eye disease?. Int. J. Environ. Res. Public Health.

[27] Mak C.M., Yik F.W.H. (2002). A study of natural ventilation in a kitchen using computational fluid dynamics (CFD). Architect. Sci. Rev..

[28] Devakumar D., Qureshi Z., Mannell J., Baruwal M., Sharma N., Rehfuess E., Saville N.M., Manandhar D.S., Osrin D. (2018). Women’s ideas about the health effects of household air pollution, developed through focus group discussions and artwork in Southern Nepal. Int. J. Environ. Res. Public Health.

[29] Edelstein M., Pitchforth E., Asres G., Silverman M., Kulkarni N. (2008). Awareness of health effects of cooking smoke among women in the Gondar region of Ethiopia: A pilot survey. BMC Int. Health Hum. Rights.

[30] Rehfuess E.A., Puzzolo E., Stanistreet D., Pope D., Bruce N.G. (2014). Enablers and barriers to large-scale uptake of improved solid fuel stoves: A systematic review. Environ. Health Perspect..

[31] Person B., Loo J.D., Owuor M., Ogange L., Jefferds M.E.D., Cohen A.L. (2012). “It is good for my family’s health and cooks food in a way that my heart loves”: Qualitative findings and implications for scaling up an improved cookstove project in rural Kenya. Int. J. Environ. Res. Public Health.

[32] Debbi S., Elisa P., Nigel B., Dan P., Eva R. (2014). Factors influencing household uptake of improved solid fuel stoves in low- and middle-income countries: A qualitative systematic review. Int. J. Environ. Res. Public Health.

[33] Loo J.D., Hyseni L., Ouda R., Koske S., Nyagol R., Sadumah I., Bashin M., Sage M., Bruce N., Pilishvili T. (2016). User perspectives of characteristics of improved cookstoves from a field evaluation in Western Kenya. Int. J. Environ. Res. Public Health.

